# The Fruits of Wampee Inhibit H_2_O_2_-Induced Apoptosis in PC12 Cells via the NF-κB Pathway and Regulation of Cellular Redox Status

**DOI:** 10.3390/molecules19067368

**Published:** 2014-06-05

**Authors:** Xiaobin Zeng, Xin Zhou, Liao Cui, Decheng Liu, Kefeng Wu, Wende Li, Ren Huang

**Affiliations:** 1 Guangdong Key Laboratory for Research and Development of Natural Drugs, Guangdong Medical College, Zhanjiang 524023, Guangdong, China; 2 Wuzhou Red Cross Hospital, Wuzhou 510663, Guangxi, China; 3 Guangdong Laboratory Animals Monitoring Institute, Guangzhou 510663, Guangdong, China

**Keywords:** *Clausena lansium*, flavanoids, neurodegeneration, apoptosis, reactive oxygen species

## Abstract

Wampee (*Clausena lansium*) fruits (CLS), whose pulp can be used to prepare fruit cups, desserts, jam, or jelly, can be eaten along with the peel. In this study, a PC12 cell model was built to observe the protective effect of CLS against H_2_O_2_-induced oxidative stress. We found that pretreatment with CLS increased cell viability and inhibited cytotoxicity, caspase-3 activity and DNA condensation. CLS also attenuated the increase in ROS production and MMP reduction. Moreover, we attempted to determine whether CLS suppressed the expression and phosphorylation of NF-κB. Western blot and immunostaining assay revealed that CLS inhibited H_2_O_2_-induced up-regulation of NF-κB p65 and pNF-κB p65. And CLS significantly suppressed the translocation of NF-κB p65 and pNF-κB p65 from cytoplasm to nuclear. Also, seven major compounds including a new flavanoid, luteolin-4'-*O*-*β*-d-gluco-pyranoside (**3**) and six known compounds **1**,**2**, **4**–**7** were isolated and identified from CLS. Their antioxidative and H_2_O_2_-induced PC12 cell apoptosis-reversing activity were determined. These findings suggest that CLS and its major constituents (flavanoids) may be potential antioxidant agents and should encourage further research into their use as a functional food for neurodegenerative diseases.

## 1. Introduction

Apoptosis, or programmed cell death, is a highly regulated process that involves the activation of a series of molecular events. Neuronal cell death due to apoptosis is a common characteristic of neurodegenerative diseases [1]. When neural cells are under oxidative stress, excessive reactive oxygen species (ROS) are produced that induce neuronal death. It has been well demonstrated that oxidative stress was associated with both physiological process of aging and pathological progression in the central nervous system (CNS) leading usually to some neurodegenerative disorders such as Parkinson’s and Alzheimer’s diseases [2,3]. Thus, a reasonable strategy for delaying the disease’s progression is to prevent reactive oxygen species (ROS) mediated cellular injury by dietary or pharmaceutical augmentation of free radical scavengers.

**Figure 1:**
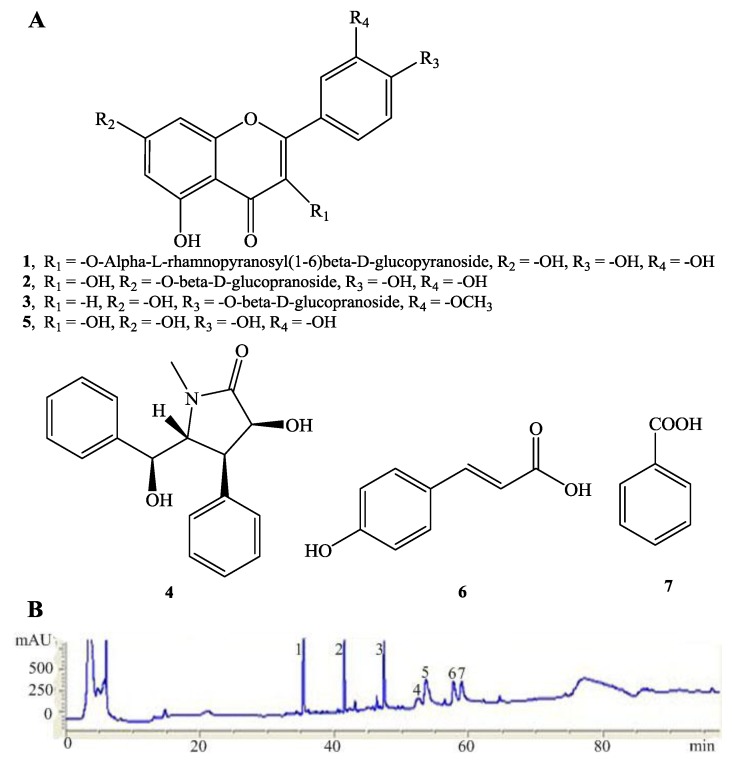
The main chemical constituents of CLS. Chemical structures of the compounds **1**–**7** (**A**). The HPLC fingerprint analysis of CLS from fruits of wampee and its main components (**B**). Compounds: rutin (**1**), quercetin-7-*O*-*α*-l-glucopranoside (**2**), luteolin-4'-*O*-*β*-d-glucopyranoside (**3**), clausenamide (**4**), quercetin (**5**), (*E*)-3-(4-hydroxyphenyl)acrylic acid (**6**) and benzoicacid (**7**).

Wampee [*Clausena lansium* (Lour.) Skeels] belongs to the Rutaceae family which is distributed widely in India, Vietnam, Thailand and southern China. Its fruits, which resemble grapes in appearance, can be eaten along with the peel at a full ripe stage. Previous studies on the bioactivities of wampee mainly focused on its leaves, stems and seeds which showed hepatoprotective activity [4], hypoglycemic [5], antifungal and antiviral activities [6], antiplatelet [7], anticancer [8], anti-inflammatory, antidiabetic and antioxidant activities [9]. It is also applied as a folk medicine in India and China for the treatment of stomachic and bronchitis, and it acts as a vermifuge as well. For the first time, this study examined the neuroprotective effects of wampee peel extracts using H_2_O_2_ at high concentration as an inducer of oxidative stress *in vitro* models [10], and further investigates its underlying mechanisms of action in H_2_O_2_-induced PC12 cells. The current findings demonstrated that CLS partly reversed the apoptosis of H_2_O_2_-induced PC12 cell via the NF-κB pathway and regulation of cellular redox status. We also investigated the constituents from CLS. Seven major compounds, including a new flavanoid, luteolin-4'-*O*-*β*-d-glucopyranoside (**3**) and six known compounds **1**,**2**, **4**–**7** were isolated from CLS and identified. The structures of these isolates **1**–**7** are shown in Figure 1A. Their antioxidative and H_2_O_2_-induced PC12 cell apoptosis reversing activity were also evaluated.

## 2. Results and Discussion

### 2.1. Structure Elucidation of the New Compound **3**

Compound **3** was obtained as an amorphous yellow powder. Positive electrospray ionization mass spectrometry (ESI-MS) gave a [M+Na]^+^ ion at *m/z* 511 and the molecular formula of C_22_H_22_O_12_ was deduced in combination with the ^1^H- and ^13^C-NMR data. The ^1^H-NMR spectrum of **3** exhibited three *meta*-coupled doublets at *δ*_H_7.96 (1H, d, *J* = 2.0 Hz, H-2'), 6.93 (1H, d, *J* = 8.4 Hz, H-5') and 7.51 (1H, dd, *J* = 8.4, 2.0 Hz, H-6') consistent with a 1',3',4'-trisubstituted flavanoid B ring. Two *meta*-coupled doublets at *δ*_H_ 6.22 (1H, d, *J* = 2.0 Hz, H-6) and 6.45 (1H, d, *J* = 2.0 Hz, H-8) were consistent with a 5,7-dioxygenated flavanoid A ring. The ^1^H-NMR spectrum of **3** displayed seven other heteroatom protons between δ_H_ 3.12 to 5.58. From the ^13^C-NMR of compound **3**, it found that there were six more carbon signals between 52.2 and 106.0 in the compound. These data suggested the presence of a glucopyranose in compound **3**. The signal of the anomeric proton appeared at 5.58 as a doublet (1H, d, *J*_1,2_ = 7.3 Hz, diaxial) and the assignments of the other glucose protons suggested this glucopyranose was a *β*-d-glucopyranose. On the basis of HMBC correlations, the singlet signal at *δ*_H_3.89 (3H, s, -OCH_3_), which typically represents a methoxy proton, showed a correlation with 147.3 (C-3'), thus confirming the assignment of the methoxy group at C-3'. From the data above, compound **3** was identified as luteolin-4'-*O*-*β*-d-glucopyranoside. The known phenolics were identified, by comparing of their spectroscopic data with data previously reported in the literature, as rutin (**1**) [11], quercetin-7-*O*-*β*-l-glucopranoside (**2**) [12], clausenamide (**4**) [13], quercetin (**5**) [12], (*E*)-3-(4-hydroxyphenyl)acrylic acid (**6**) [14] and benzoic acid (**7**).

### 2.2. Analysis of the Constituents of CLS and Quantitation of Lutin and Quercetin-7-O-β-l-gluco- pyranoside in CLS

The TP and TF content of the CLS were determined to be 75.6 ± 8.7 mg/g gallic acid equivalents and 34.2 ± 3.0 mg/g rutin equivalents, respectively. The HPLC fingerprint analysis of the active fraction is shown in Figure 1B, where seven compounds, namely rutin (**1**), quercetin-7-*O*-*α*-l-glucopyranoside (**2**), luteolin-4'-*O*-*β*-d-glucopyranoside (**3**), clausenamide (**4**), quercetin (**5**), (*E*)-3-(4-hydroxyphenyl)acrylic acid (**6**) and benzoic acid (**7**) were identified. The contents of rutin and quercetin-7-*O*-*β*-l-glucopyranoside in CLS were also measured by HPLC to be 3.21‰ ± 0.23‰ and 2.80‰ ± 0.18‰, respectively.

### 2.3. Antioxidative Activity of CLS and Its Isolates **1**–**6**

The DPPH radical scavenging activity of CLS and its isolates **1**–**6** increased with increased concentration and the IC_50_ (the concentration required to scavenge 50% of radical) values of CLS and positive control (gallic acid) were 18.88 ± 1.23 and 19.31 ± 1.07 µg/mL, respectively. Gallic acid, as a trihydroxybenzoic acid, is used as a standard for determining the phenol content of various analytes by the Folin-Ciocalteau assay, acts as an antioxidant and also helps to protect human cells against oxidative damage, so we used gallic acid as positive control.

**Figure 2:**
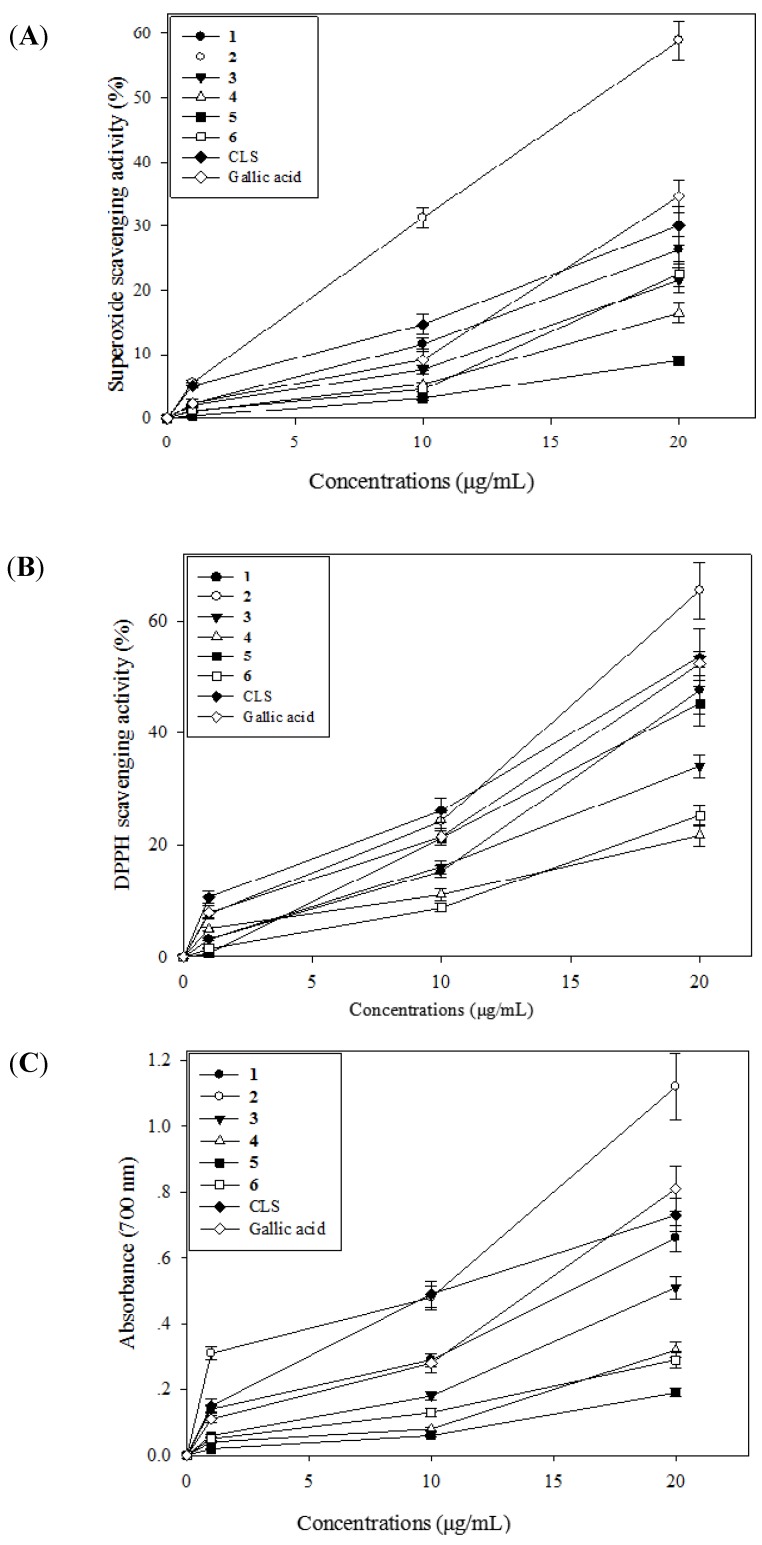
Antioxidant activity of CLS from the fruits of wampee peel. Superoxide anion scavenging activity of CLS (**A**); DPPH radical scavenging activity of CLS (**B**); Reducing power activity of CLS (**C**).

As shown in Figure 2A, the isolate **2** and CLS exhibited comparable activity level compared with positive control. The isolate **2** demonstrated a dose-dependent increase in DPPH radical scavenging activity by 7.56%, 24.05% and 65.45% at the concentrations of 1, 10 and 20 μg/mL, as compared to the control. The superoxide anion scavenging effects of CLS and the isolates 1–6 were analyzed and the results are given in Figure 2B. The isolates **1**,**2** and CLS exhibited comparable activity level compared with positive control (Gallic acid). The isolate **2** demonstrated the strongest superoxide anion scavenging activity by 5.62%, 31.31% and 58.92% at the concentrations of 1, 10 and 20 μg/mL, as compared to the control. Reducing power is widely used in evaluating the antioxidant activity of plant polyphenols. The reducing power is generally associated with the presence of reductones, which exert antioxidant action by breaking the free radical chains by donating a hydrogen atom. In the present study, the CLS and the isolates **1**–**6** exhibited a strong reducing power, as shown in Figure 2C. At 20 μg/mL, the reducing power ability of the isolate **2** and CLS were 1.12 ± 0.10 and 0.73 ± 0.06, respectively. While, the reducing power ability of positive control (gallic acid) was 0.81 ± 0.07. All in all, the CLS and its isolates showed the antioxidative activity and reducing power, and these activities of the CLS are probably due to the presence of phenolic compounds, especially flavanoids, which might act as electron donors.

### 2.4. Effects of CLS and its Isolates **1**–**6** on Cell Viability in PC12 Cells

**Figure 3:**
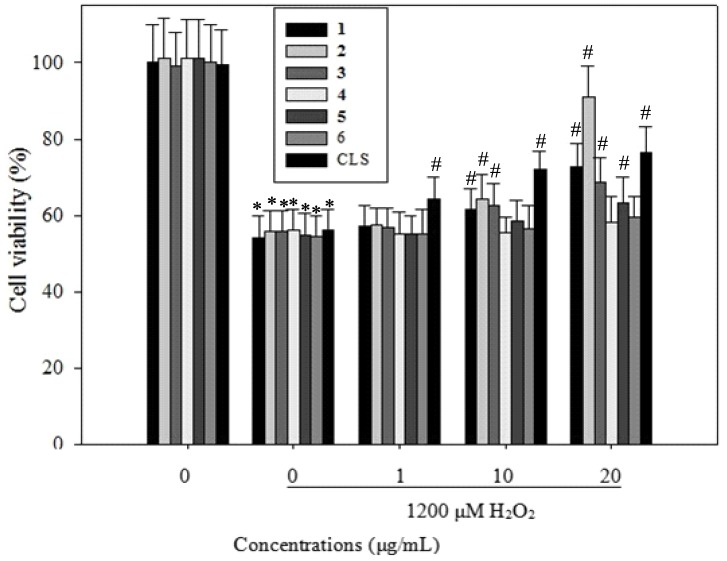
PC12 Cells were pretreated with the indicated concentrations of CLS for 12 h, then further co-cultured with 1,200 µM H_2_O_2_ for 6 h. Cell viability were determined by MTT assays, and expressed as percentages of the corresponding values for the control group.

Note: *p < 0.05 *vs.* control; *^# ^p* < 0.05 *vs.* 1200 μM H_2_O_2_.

We next sought to determine whether CLS and the isolates **1**–**6** can reverse the apoptosis of H_2_O_2_-induced PC12 cells. The viability of PC12 cells incubated with 1,200 µM H_2_O_2_ for 24 h was 55% of the control value (Figure 3), but this increased significantly by 9.12%, 17.13%, 21.26% when cells were pretreated with CLS at 1, 10 and 20 µg/mL. As shown in Figure 3, 6 h after treatment, the isolate **1** showed a dose-dependent increase in the viability of PC12 cells by 2.13%, 6.59% and 17.56% at the concentrations of 1, 10 and 20 μg/mL, respectively, as compared to the model. Isolate **2** demonstrated a dose-dependent increase in the viability of PC12 cells by 2.57%, 9.34% and 35.91% at the concentrations of 1, 10 and 20 μg/mL, respectively, as compared to the model. Isolate **3** significantly increased the viability of PC12 cells by 1.84%, 7.53% and 13.52% at concentrations of 1, 10 and 20 μg/mL, as compared to the model. Isolate **5** significantly increased the viability of PC12 cells by 3.55% and 8.24% at concentrations of 10 and 20 μg/mL, as compared to the model. These results suggested that CLS and its isolates **1**–**3**, **5** could promote the viability of H_2_O_2_-induced PC12 cells against apoptosis.

### 2.5. Effects of CLS on DNA Condensation, Intracellular ROS Production and MMP Loss in PC12 Cells

**Figure 4:**
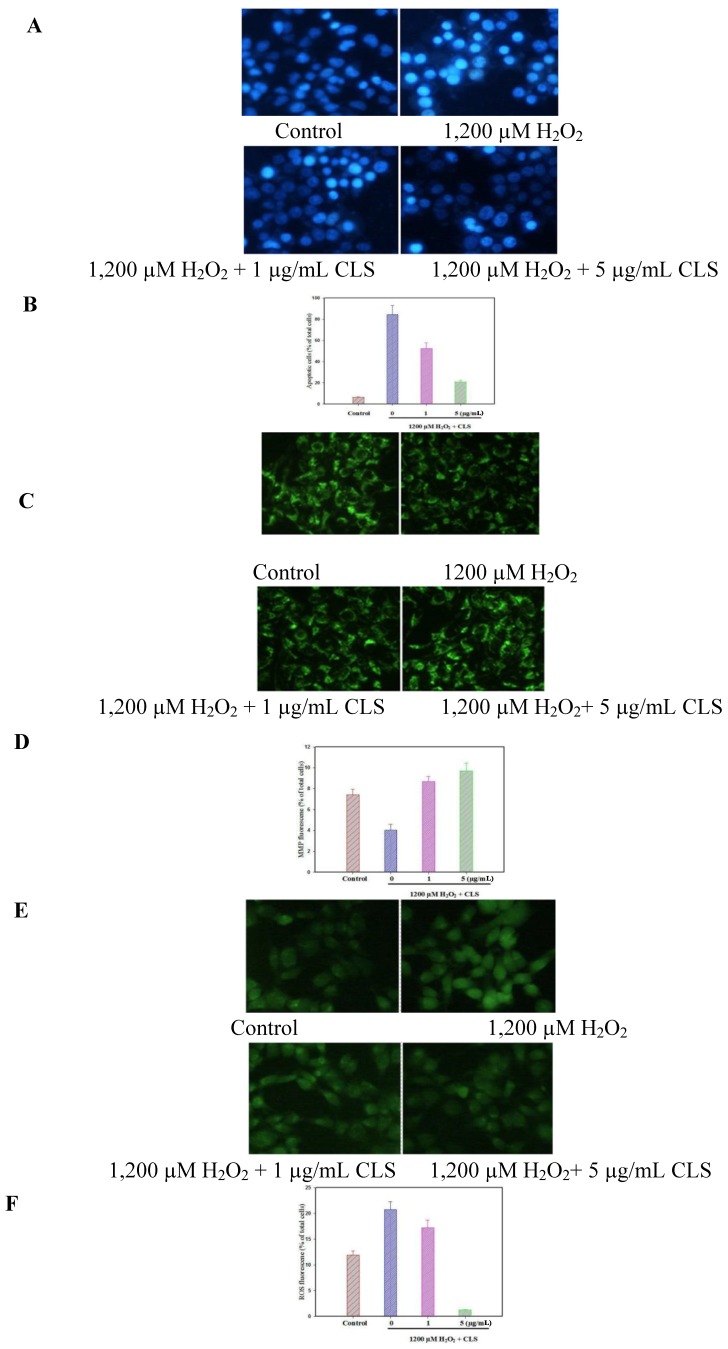
Apoptotic nuclei were measured by incubation of the cells with DAPI fluorescence dye followed by observation under fluorescence microscopy (20×). Morphology of apoptotic cells stained with DAPI (**A**) and the ratio of apoptotic cells (**B**); Effects of CLS on intracellular ROS production (**C** and **D**): Cells were pretreated with the indicated concentrations of CLS for 12 h, and then co-cultured further with 1,200 µM H_2_O_2_ for 5 h, respectively. Then, ROS accumulation was measured by incubation of the cells with DCFH-DA fluorescent dye followed by observation under fluorescence spectrophotometer (20×); Effects of CLS on intracellular loss of MMP (**E** and **F**) in PC12 cells. Cells were pretreated with the indicated concentrations of CLS for 12 h, and then co-cultured further with 1,200 µM H_2_O_2_ for 5 h, respectively. Then, MMP reduction was measured by incubation of the cells with rhodamine 123 fluorescent dye followed by observation under fluorescencespectrophotometer (20×). The images shown are representative of three experiments. Data are presented as the means ± S.E.M. (*n* = 5).

DAPI staining revealed that nuclear DNA condensation and nuclear fragmentation occurred after treatment with 1,200 µM of H_2_O_2_. Pretreatment with CLS inhibited these apoptotic features (Figure 4A). The ratio of apoptotic cells incubated with 1,200 µM H_2_O_2_ for 24 h was 84.45% while the negative control value was 6.38% (Figure 4B), but this decreased significantly to 52.38% and 20.83% when cells were pretreated with CLS at 1 and 5 µg/mL (*p* < 0.001 and *p* < 0.001, respectively). These results indicate that CLS has an anti-apoptotic effect against H_2_O_2_-induced apoptosis in PC12 cells. Treatment with 1,200 µM H_2_O_2_ significantly increased intracellular ROS production from 11.89% (normal cells) to 20.7% of total cells, but CLS at 1 and 5 µg/mL significantly reduced ROS production to 17.17% and 1.22% of total cells (*p* < 0.05, and *p* < 0.001, respectively) (Figure 4D). Besides, treatment with 1,200 µM H_2_O_2_ significantly decreased the fluorescence intensity of the MMP probe from 7.41% (normal cells) to 4.02% of total cells. However, CLS pretreatment at 1 and 5 µg/mL maintained MMP at 8.67% and 9.72% of total cells (*p* < 0.05, and *p* < 0.05, respectively) (Figure 4F).

Meanwhile, treatment with 1,200 µM H_2_O_2_ significantly increased ROS fluorescence intensity compared with control image, but CLS significantly inhibited ROS fluorescence intensity compared with H_2_O_2_-treated image (Figure 4C). On the other hand, treatment with 1,200 µM H_2_O_2_ significantly decreased MMP fluorescence intensity compared with the control, but CLS significantly increased MMP fluorescence intensity compared with H_2_O_2_-treated group (Figure 4E).

### 2.6. Effects of CLS on the Pathway of NF-κB

Nuclear factor kappa B (NF-κB), a redox-sensitive transcription factor, is regulated by various apoptotic stimuli or inhibitors. It is involved in brain function, particularly following injury and in neurodegenerative conditions such as Alzheimer’s disease. A number of reports have shown that NF-κB is inhibited by apoptosis-inducing agents in human cancer cells. NF-κB itself may serve as a pro-survival agent in various circumstances. The activation of NF-κB is known to induce the expression of Bcl-2 and Bcl-xL. Inducible loss of NF-κB activity is associated with the down-regulation of anti-apoptotic Bcl-2 family members and the occurrence of apoptosis. NF-κB is composed of homo- and heterodimeric complexes of members of the Rel family: p50, p65, c-Rel, p52 and RelB. And the most common and best-characterized form of NF-κB is the p65/p50 heterodimer. So we mainly detected the NF-κB p65 in this manuscript.

**Figure 5:**
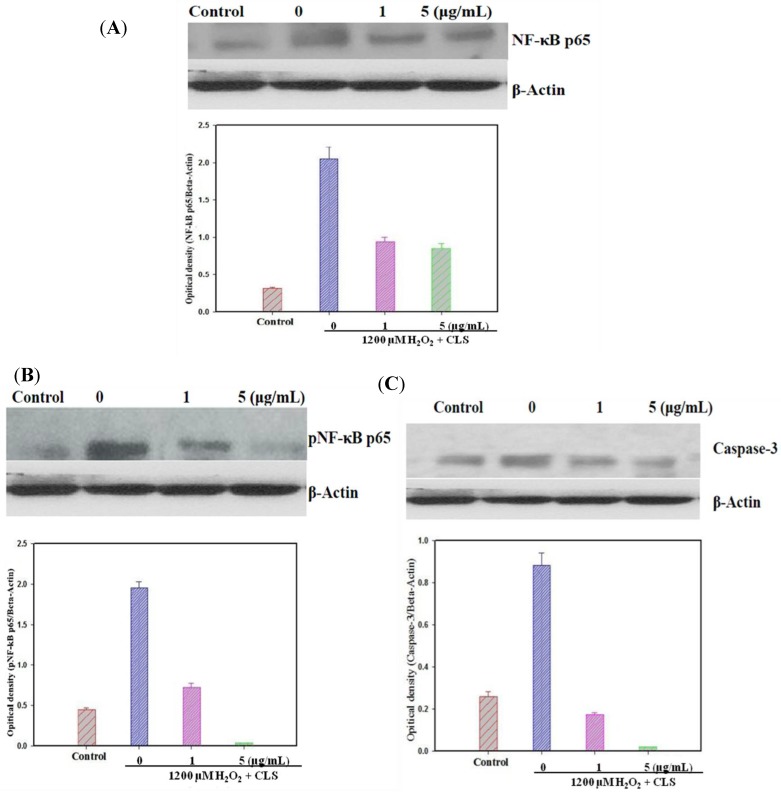
Effects of CLS on NF-κB p65, pNF-κB p65 and caspases-3 expression in H_2_O_2_-treated PC12 cells by western blot analysis. Densitometric analyses of protein bands were normalized to a loading control *β*-actin. Data are presented as the means ±S.E.M. (*n* = 5). All experiments included in vehicle, 1200 µM and CLS treatment using *β*-actin as the loading control. (**A**) Expression of NF-κB p65, (**B**) expression of pNF-κB p65, and (**C**) expression of caspases-3.

**Figure 6:**
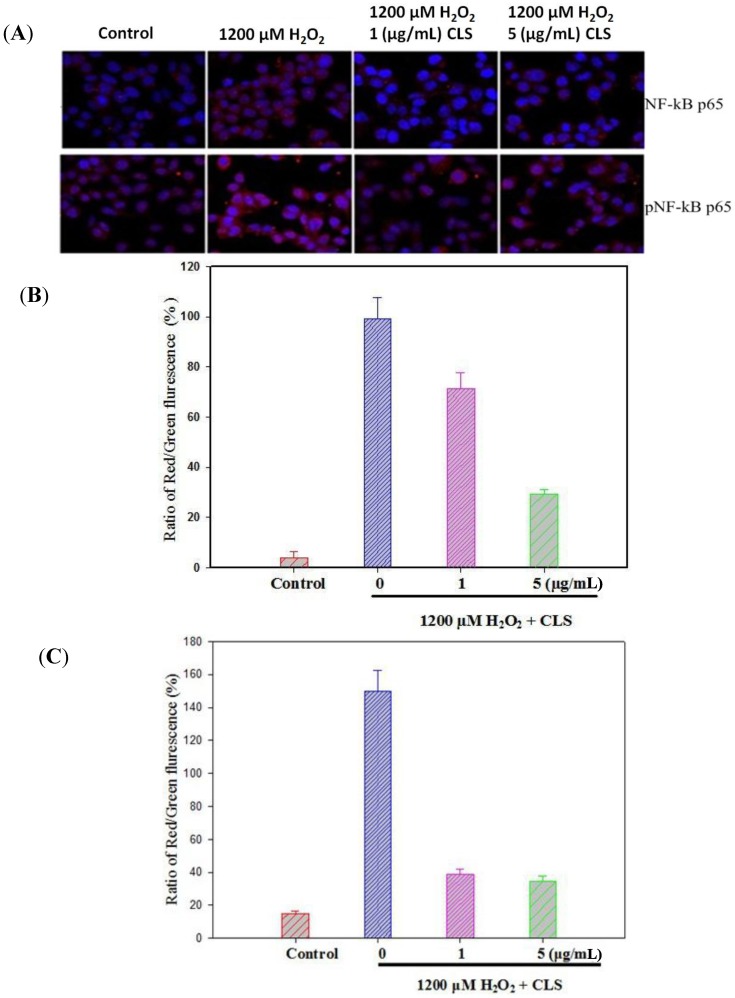
Effects of CLS on nuclear translocation of NF-κB p65 (**A** upper and **B**) and pNF-κB p65 (**A** bottom and **C**) in PC12 cells. NF-κB p65 and pNF-κB p65 labeled with Alexa-488 (red fluorescence), nuclear stained with DAPI (blue fluorescence) and merge (red and blue fluorescence). Magnification 20×. The images shown are representative of three experiments. Data are presented as the means ±S.E.M. (*n* = 5).

NF-κB p65 levels were measured in PC12 cells by western blot. As shown in Figure 5A, CLS significantly decreased the expression of NF-κB p65 in a concentration-dependent manner. Nuclei stained with DAPI appear in blue, and NF-κB p65 labeled with Alexa 488 in red. Treatment with 1,200 µM H_2_O_2_ significantly stimulated entry of NF-κB p65 to the nucleus from the cytoplasm compared with control image. However, CLS (1 and 5 μg/mL) could obviously suppress NF-κB p65 entry to the nucleus from the cytoplasm (Figure 6A,B).

pNF-κB p65 levels were measured in PC12 cells by western blot. As shown in Figure 5B, CLS significantly decreased the expression of pNF-κB p65 in a concentration-dependent manner. Moreover, activation of NF-κB p65 pathway was observed by immunostaining. Nuclear stained with DAPI in blue, and pNF-κB p65 labeled with Alexa 488 in red. Treatment with 1200 µM H_2_O_2_ significantly stimulate pNF-κB p65 entering nuclear from cytoplasm compare with control image. However, CLS (1 and 5 μg/mL) could obviously suppress pNF-κB p65 entering nuclear from cytoplasm (Figure 6A,C).

### 2.7. Effects of CLS on Caspase-3 Expression

To further examine the participation of the mitochondrial apoptotic pathway in the neuroprotective activity of CLS against H_2_O_2_-induced PC12 cells, the activation of caspase-3 was also detected. As shown in Figure 5C, the caspase-3 activity up-regulated in the H_2_O_2_-treated group compared with the control, whereas CLS (1 and 5 μg/mL) pretreatment caused a significant decrease in caspase-3 activity.

## 3. Experimental

### 3.1. General Information

Optical rotations were measured using a JASCO P-1030 (Tokyo, Japan) automatic digital polarimeter. NMR spectra (400 MHz for ^1^H-NMR, 100 MHz for ^13^C-NMR), were recorded on a Bruker DPX-400 spectrometer Karlsruhe, Germany) using standard Bruker pulse programs. Chemical shifts were showed as the *δ*-value with reference to tetramethylsilane (TMS) as an internal standard. And ESI-MS data were obtained on an Agilent 1200 HPLC/6410B TripleQuad mass spectrometer (Bremen, Germany). Sephadex LH-20 (Pharmacia, Sweden), silica gel (Qingdao Ocean Chemical Co., Ltd, Qingdao, China), Octadecylsilanized (ODS) silica gel (Macherey-Nagel, Duren, Germany) were used for column chromatography. TLC was carried out on Silica gel 60 F_254_ (0.25 mm, Merck, Darmstadt, Germany), and RP-18 F_254_ (0.25 mm, Merck) plates, and spots were visualized by spraying with 15% H_2_SO_4 _followed by heating. HPLC was performed using an Phenomenex C_18_ column (Ø 250 mm × 4.6 mm).Dulbecco’s modified Eagle’s medium (DMEM), fetal bovine serum (FBS), and trypsin-EDTA solution (1×) were obtained from Hyclone (Logan, UT, USA). Vitamin E (VE) and gallic acid (GA) were purchased from the National Institute for the Control of Pharmaceutical and Biological Products (Beijing, China). Α,α-Diphenyl-β-picrylhydrazyl (DPPH), NADH, PMS and NBT were purchased from Sigma Chemical Co. (St. Louis, MO, USA). All other chemicals used for analysis were AnalaR grade, obtained from China Medicine (Group) Shanghai Chemical Reagent Corporation (Shanghai, China).

### 3.2. Plant Material

Fresh mature fruits from *Clausena lansium* (Lour.) Skeels were collected in June 2011 from rural areas around Yunfu, Guangdong Province, Central China, and characterized by the corresponding author. A voucher specimen was deposited at the herbarium of Guangdong Key Laboratory for Research and Development of Natural Drugs, Guangdong Medical College, China. The plant material was air dried indoors at room temperature.

### 3.3. Extraction and Isolation

Air dried, the fruits of *Clausena lansium* (Lour.) Skeels (5.0 kg) were extracted in 95% ethanol (20.0 L) at 40 °C for 24 h. The extracts were filtered through qualitative filter paper with 30–50 µm pore size (DX102, Xinhua Paper Co., Ltd., Hangzhou, China). Evaporation of the organic solvent under a vacuum at 55 °C yielded a crude extract (CLS, 1.65 kg). The concentrated brown syrup was resuspended in water and gradually partitioned with petroleum ether (3 L × 3), ethyl acetate (3 L × 3) and water-saturated *n*-butanol (3 L × 3) to afford 120.0 g, 235.3 g and 330.5 g of dried organic extracts, respectively. The ethyl acetate fraction with the most potential activity was fractionated over a silica gel (200~300 mesh) column eluting with increasing amounts of MeOH in CHCl_3_ to give 10 fractions. The CHCl_3_-MeOH (25:1) elution was further purified on a silica gel column and eluted with CHCl_3_/MeOH (100:1→5:1), yielding compounds **3** (26.08 mg) and **6** (34.53 mg). The CHCl_3_-MeOH (20:1) eluate was further purified by a silica gel column and eluted with CHCl_3_/MeOH (100:1→2:1), yielding compounds **2** (43.88 mg) and **7** (48.12 mg). The CHCl_3_-MeOH (15:1) eluate was subjected to silica gel and Sephadex LH-20 column chromatography, followed by an octadecylsilanized silica gel (ODS) column eluted with MeOH/H_2_O (10: 90-100: 0) to give phenolics **1** (25.07 mg) and **4** (89.47 mg). The CHCl_3_-MeOH (10:1) eluate was subjected to ODS column chromatography, followed by a Sephadex LH-20 column with CHCl_3_/MeOH (7:3), yielding compound **5** (108.49 mg). The structures of phenolics **1**-**7** are shown in Figure 1.

### 3.4. Luteolin-4'-O-β-d-glucopyranoside (**3**)

Amorphous yellow powder; ESI-MS (positive-ion mode) [M+Na]^+^*m/z* 511. ^1^H-NMR (DMSO-*d*_6_) *δ*_H_: 3.12-5.41 (sugar protons), 3.89 (3H, s, -OCH_3_), 5.58 (1H, d, *J* = 7.3 Hz, H-1''), 6.22 (1H, d, *J* = 2.0 Hz, H-6), 6.45(1H, d, *J* = 2.0 Hz, H-8), 6.93 (1H, d, *J* = 8.4 Hz, H-5'), 7.51 (1H, dd, *J* = 8.4, 2.0 Hz, H-6'), 7.96 (1H, d, *J* = 2.0 Hz, H-2'), 9.82 (1H, s, 3'-OH), 10.89 (1H, s, 7-OH), 12.63 (1H, s, 5-OH).^13^C-NMR (DMSO-*d*_6_) *δ*_C_: 156.8 (C-2), 133.4 (C-3), 177.9 (C-4), 161.7 (C-5), 99.2 (C-6), 164.6 (C-7), 94.2 (C-8), 156.8 (C-9), 104.5 (C-10), 122.5 (C-1'), 115.7 (C-2'), 147.3 (C-3'), 149.8 (C-4'), 113.9 (C-5'), 121.5 (C-6'), 101.5 (C-1''), 74.8 (C-2''), 77.9 (C-3''), 70.2 (C-4''), 76.8 (C-5''), 61.0 (C-6''), 56.1 (3'-OCH_3_).

### 3.5. Analysis of Total Phenolic Content and Total Flavonoid Content

Total phenolic (TP) content of CLS was determined using the Folin-Ciocalteau assay according to a previously described method [15]. Total flavonoid (TF) content of CLS was determined by a colorimetric assay described earlier [16].

### 3.6. HPLC Analysis and Quantitation of Lutin and Quercetin-7-O-β-l-glucopranoside in CLS

HPLC was performed to analyze the compounds in the fruits of *Clausena lansium* (Lour.) Skeels on a Phenomenex C_18_ column (Ø 250 mm × 4.6 mm) on an Agilent series 1200 instrument (Santa Clara, California, USA) under following conditions: mobile phase: H_2_O (A), MeOH (B); elution program: linear gradient from 10% B to 15% B in 20 min, 15% B to 70% B in 20 min, 70% B to 80% B in 20 min and then 100% B maintained for 20 min; flow rate: 0.80 mL/min; detection wavelength: 210 nm; injection volume: 10 µL; and oven temperature: 24 °C.

### 3.7. Determination of in Vitro Antioxidant Activity

The free radical-scavenging activity of CLS was measured using DPPH according to the procedure described by Yen and Chen [17]. The ability of CLS to scavenge superoxide radical was assayed by the NBT reduction method according to a described procedure [18] with slight modifications. The reaction mixture used for the O_2_^−^ scavenging activity assay containing Tris-HCl (pH 8.1, 50 mM, 222.5 μL), NADH (0.15 mM, 125 μL) , PMS (0.03 mM, 25 μL), NBT (0.10 mM, 125 μL) and compound solution (2.5 μL), in the final volume of 500 μL. All components were dissolved in Tris-HCl 50 mM, pH 8.1. The reaction was conducted at 37 °C for 5 min, and initiated by the addition of PMS. The absorbance of the resulting solution was measured spectrophotometrically at 570 nm. The reducing power was determined by the method of [8]. The antioxidant activity of vitamin E was determined for comparison.

### 3.8. Cell Culture

PC12 cells were obtained from Peking Union Medical College (Beijing, China) and were cultured in DMEM medium supplemented with 10% heat-inactivated FBS, 100 U/mL penicillin and 100 μg/mL streptomycin at 37 °C in a humidified atmosphere of 5% CO_2_ and 95% air.

### 3.9. Cell Viability Assay

Cell viability was determined by using the MTT assay, based on the conversion of MTT to formazan crystals by mitochondrial dehydrogenases [19]. Briefly, PC12 cells were seeded at a density of 1 × 10^4^ cells/well in 96-well micro-plates for 24 h. After incubation, PC12 cells were pretreated with various doses of CLS (1 and 5 μg/mL) for 12 h. To test the protective effect of CLS against H_2_O_2_-induced neurotoxicity, the PC12 cells were co-incubated with 1,200 μM of H_2_O_2_ and different concentrations of CLS and the isolates (1 and 5 μg/mL) for 5 h. At the end of treatment, Cell viability was performed according to a reported protocol [20]. Cell viability was expressed as percentage of non-treated control.

### 3.10. DAPI Staining Analysis

To detect apoptosis, nuclear staining was done [21]. Briefly, PC12 cells were seeded at a density of 5 × 10^4^ cells/well in a six-well plate. 24 h after the seeding, PC12 cells were pretreated with various doses of CLS (1 and 5 μg/mL) for 12 h and then co-cultured with H_2_O_2_ for 5 h. After treatment, the cells were washed three times with ice-cold PBS, then cells were stained with DAPI (Roche, Basel, Switzerland) solution and analyzed with a fluorescence microscope. Apoptotic cells were identified by morphologic changes (condensation and fragmentation of their nuclei). Images were obtained with fluorescent microscopy on an TE-2000E microscope (Nikon, Tokyo, Japan). Images were processed using Photoshop software (Adobe, San Jose, CA, USA).

### 3.11. Measurement of Intracellular ROS Accumulation

DCFH-DA can be deacetylated in cells, where it can react quantitatively with intracellular oxidants (mainly H_2_O_2_) to form the fluorescent product, DCF, which is retained within the cells. PC12 cells (1 × 10^5^) were cultured in 6-well plates for 24 h, followed by pretreatment with various doses of CLS (1 and 5 μg/mL) for 12 h and then co-cultured with H_2_O_2_ for 5 h. The cells were rinsed with PBS solution, and then treated with 15 mM DCFH-DA. After incubation for 30 min at 37 °C, cells were examined at 530 nm with a fluorescence spectrophotometer (LeicaDMI 6000B, Leica, Solms, Germany), with excitation at 488 nm. DCFH-DA fluorescence images data were then observed using a fluorescence microscope (20×).

### 3.12. Measurement of Intracellular Mitochondrial Membrane Potential (MMP)

MMP was monitored using the fluorescent dye rhodamine 123. This cell-permeable cationic dye preferentially partitions into the mitochondria based on the highly negative MMP. PC12 cells (5 × 10^4^) were cultured in 6-well black plates for 24 h, and then pretreated with various doses of CLS (1 and 5 μg/mL) for 12 h and then co-cultured with H_2_O_2_ for 5 h. The cells were rinsed with PBS solution, and 1 mM rhodamine 123 (Sigma, St. Louis, MO, USA) was added to the wells. After incubation at 37 °C for 30 min, the cells were examined at 530 nm with a fluorescence spectrophotometer, with excitation at 480 nm. Rhodamine123 fluorescence images data were then observed using a fluorescence microscope (20×).

### 3.13. Immunostaining Assay

PC12 were cultured in 24-well plates at 5 × 10^4^ cells/well and then pretreated with various doses of CLS (1 and 5 μg/mL) for 12 h and then co-cultured with H_2_O_2_ for 5 h. After fixing with 95% ethanol for 15 min on ice, cells were permeabilized with 0.5% Triton for 3 min at room temperature and then blocked with 1% bovine serum albumin solution for 1 h. Then cells were exposed to NF-κB-p65 or pNF-κB-p65 subunit antibody for 1 h followed by Alexa-488 conjugated secondary antibody for 1 h at room temperature. Subsequently, nuclei were stained with DAPI for another 10 min at room temperature in the dark. The nuclear translocation of NF-κB-p65 or pNF-κB-p65 was visualized by an IN Cell 2000 Image system with a 400× lens (GE Healthcare Limited, Buckinghamshire, UK).

### 3.14. Western Blot Analysis

PC12 cells were seeded at density of 2 × 10^6^ cells in a 25-cm^2^ flask for 24 h. After incubation, cells were pretreated with various doses of CLS (1 and 5 μg/mL) for 12 h and then co-cultured with H_2_O_2_ for 5 h. Cells were collected and lysed on ice, then cell lysates were clarified by centrifugation and then the supernatants were collected and stored at −70 °C until use.

Protein concentrations were measured using the Bradford method [22]. An equal amount of protein was loaded and separated using 10% polyacrylamide gel electrophoresis and transferred onto polyvinylidene fluoride membrane. After blocking the nonspecifc site with 5% non-fat dried milk in 50 mM Tris-buffered saline containing 0.1% Tween-20 (TBST) for 1 h at room temperature, the membrane was then incubated with the specific primary antibody (1:500) at 4 °C overnight. Following three washes with TBST, the blots were incubated with the secondary horseradish peroxidase-conjugated goat anti-rabbit IgG antibody (1:1000) for 1 h at room temperature. Subsequently, the blots were washed again for three times with TBST and then visualized by enhanced chemiluminescence (ECL) kit according to the manufacturer’s instruction. The band densities were quantified from three different observations using an ImageJ software (National Institutes of Health, Bethesda, MD, USA).

### 3.15. Statistical Analysis

All data were expressed as mean ± S.D. from at least three independent experiments, each performed in quintuplicate. Statistical significance was determined by analysis of variance and subsequently applying the Dunnett’s *t*-test (*p* < 0.05).

## 4. Conclusions

Apoptosis might occur in, and contribute to the onset and progression of neurodegenerative disorders [23]. Different factors have been suggested as stimulators of apoptotic pathways. Among them, dysregulation of homeostasis between generation and quenching of free radicals has a great importance. Researchers have made considerable efforts to search for candidates capable of modifying this imbalance and in favor of removing excess free radicals or suppressing their generation thus maintaining cell integrity. Interestingly, free radicals not only cause damage to cellular structures, but also provoke cellular protective responses in vulnerable neurons by the compensatory upregulation of antioxidant enzymes and activation of oxidative sensitive factors, for example NF-κB [24]. In the current study, we examined the protective effect of CLS against H_2_O_2_-induced cytotoxicity in PC12 cells.

The PC12 cell line, derived from a pheochromocytoma of the rat adrenal medulla, is a useful model system for the study of numerous problems in neurobiology and neurochemistry [25]. Recent studies also showed that ROS are deeply involved in the pathophysiology of several neurodegenerative diseases, such as Alzheimer’s disease [26] and PD [27]. ROS can affect mitochondrial function through the mitochondrial ATP-sensitive potassium (mito K_ATP_) channels and the mitochondrial permeability transition pore (mPTP) [28]. On the other hand, maintenance of MMP is necessary for production of energy ATP and preservation of cellular homeostasis. Akao *et al.* have shown that maintenance of MMP is a critical primary determinant of cell survival [29]. Tang *et al.* also reported that oxidative stress by H_2_O_2_ exposure led to apoptosis, ROS increasing and MMP loss of PC12 cells [30].

Nuclear factor kappa-light-chain-enhancer of activated B cells (NF-κB) is a protein complex that controls the transcription of DNA. NF-κB is found in almost all animal cell types and is involved in cellular responses to stimuli such as stress, free radicals, cytokines, and ultraviolet irradiation. Incorrect regulation of NF-κB has been linked to cancer, septic shock, viral infection, and improper immune development [31,32,33,34,35]. NF-κB is widely used by eukaryotic cells as a regulator of genes that control cell proliferation and cell survival. Active NF-κB turns on the expression of genes that keep the cell proliferating and protect the cell from conditions that would otherwise cause it to die via apoptosis. Antioxidants, which have been widely used for many years to inhibit NF-κB, were reported to block activation of NF-κB by various stimuli [36] or directly preventing NF-κB binding to DNA [37]. It suggests that ROS could be ubiquitous mediators of NF-κB activation. NF-κB is sensitive to variations of the cellular redox potential, but most of the observed inhibitory actions were due to the numerous side effects of antioxidants on multiple cellular signaling pathways.

The caspase-3 protein is a member of the cysteine-aspartic acid protease (caspase) family [38]. Sequential activation of caspases plays a central role in the execution-phase of cell apoptosis. Caspases exist as inactive proenzymes that undergo proteolytic processing at conserved aspartic residues to produce two subunits, large and small, that dimerize to form the active enzyme. Caspase-3 cleaves and activates caspases 6 and 7. The protein itself is processed and activated by caspases 8, 9, and 10. It is the predominant caspase involved in the cleavage of amyloid-beta 4A precursor protein, which is associated with neuronal death in Alzheimer’s disease. Alternative splicing of this gene results in two transcript variants that encode the same protein. Caspase-3 has been found to be necessary for normal brain development as well as its typical role in apoptosis, where it is responsible for chromatin condensation and DNA fragmentation [39].

In the present study, our results revealed that high concentrations of H_2_O_2_ markedly decreased the cell viability, obviously increased the number of apoptotic cells in the DAPI staining, confirming its neurotoxicity in PC12 cells. In this *in vitro* model, on pretreatment with CLS (1 and 5 μg/mL) in presence of 1,200 μM H_2_O_2_, the changes of PC12 cells induced by H_2_O_2_ were partly reversed. To further characterize the mechanism of CLS on functions of PC12 cells, CLS significantly inhibited the expression and phosphorylation of NF-κB, and translocation of NF-κB from cytoplasm to nuclear where NF-κB could bind to consensus sequence on the promoter and consequently enhance the expression of related enzymes and cytokines.

In conclusion, this study revealed the potential neuroprotective targets of CLS (e.g., inhibition of cytotoxicity, reduction of ROS accumulation, DNA condensation, MMP stabilization, inhibition of caspase-3 activity, inhibition of NF-κB expression and phosphorylation, suppression of NF-κB translocation from cytoplasm to nuclear ). The neuroprotective activity of CLS may be due to its free radical-scavenging and antioxidant activity, resulting from the presence of flavanoids in the extracts. Also, seven major compounds, including a new flavanoid, luteolin-4'-*O*-*β*-d-glucopyranoside (**3**) and six known compounds **1**,**2**, **4**–**7** were isolated from CLS and identified. Their antioxidative and H_2_O_2_-induced PC12 cell apoptosis-reversing activity were determined. The neuroprotective action of CLS may potentially be applied in the treatment of neurodegenerative diseases such as AD.
